# The Relationship between Economic Growth and Air Pollution—A Regional Comparison between China and South Korea

**DOI:** 10.3390/ijerph17082761

**Published:** 2020-04-16

**Authors:** Min Jiang, Euijune Kim, Youngjin Woo

**Affiliations:** Department of Agricultural Economics and Rural Development and Research Institute of Agricultural and Life Sciences, Seoul National University, Seoul 08826, Korea; minmin@snu.ac.kr (M.J.); alternative@snu.ac.kr (Y.W.)

**Keywords:** air pollution, regional economic growth, international comparison, China, South Korea

## Abstract

This paper analyzes the interaction between regional economic growth and air pollution in China and Korea. The relationship between gross regional product per capita and industrial emission of sulfur dioxide emission is examined at the regional level using simultaneous equation models covering 286 cities in China and 228 cities and counties in South Korea of the period 2006–2016. The results find that regional differences existed in the relationship between air pollution and economic growth in two countries. In both countries, an inverted U-shaped pattern was found in metropolitan areas while a U-shaped pattern of non-metropolitan areas. Although the emissions of pollutants in metropolitan areas of both countries have shown a downward trend in recent years, there is still a large gap between the overall emission levels of China and South Korea. Moreover, the level of pollutant emissions of China’s metropolitan areas is much higher than in non-metropolitan areas, while the opposite result has occurred in Korea. In China, there was an inverted U-shaped relationship of the eastern and northwest region, while U-shaped relationships existed in the southwest, central and northeast regions.

## 1. Introduction

The relationship between economic growth and environmental pollution has been a longstanding global concern since the 1970s. According to the Environmental Kuznets Curve (EKC) theory first proposed by Grossman and Krueger [1] and Panayotou [2], there is an inverted u-shaped relationship between income level and environmental degradation. In the first stage, economic growth is associated with environmental deterioration. At this stage, the increase in industrial activity in countries with lower levels of economic development leads to an increase in energy-intensive production and an increase in pollutant emissions. With the development of services and knowledge-based technology-intensive industries, the environmental degradation trend gradually declines due to environmental awareness, coupled with changes in production and stricter environmental regulations. The EKC literature suggests that economic growth may affect environmental welfare through three different channels: scale effects, composition effects and technique effects. The growth of the economic scale would result in a proportional growth in environmental pollution, and the changes in the industrial structure would lead to the reduction of pollution intensity [3]. Further economic growth causes technological progress through which dirty and obsolete technologies are replaced by upgraded and cleaner technologies that improve environmental quality [4]. Extensive previous works found that the impacts of economic growth on environmental pollution can be generally divided into four relationships: an inverted U-shaped relationship [1], a monotonically increasing relationship [5,6,7,8,9,10], a U-shaped relationship [4,9,11,12,13,14] and a N-shaped relationship [1,15,16,17]. However, the reverse effect of the environment on economic growth has received relatively little attention. Another limitation of existing research is that most of the previous research discussed the relationship between economic growth and environmental pollution from the national level viewpoint, with little attention paid to regional perspectives.

Clarifying the relationship between environmental pollution and economic growth at the regional level is critical for two reasons. First, the regional economic gap is a key obstacle to effective control of pollutant emissions because different economic conditions and industrial structures are closely related to local emission patterns and trends. The regional economic gap determines different economic growth patterns and industrial structures. For instance, Beijing, the capital city and a first-tier city in east China, had a relatively high level of gross regional product (GRP) per capita of US$ 19,769 in 2018. The service industry was highly developed, accounting for 81.0% of the total GRP, and the manufacturing industry only accounted for 18.6%. In the three typical third-tier cities of east, west and central China, namely Shantou city, Xianyang city, and Chenzhou city, the per capita GRPs in 2018 were US$ 6308, US$ 8261 and US$ 7182, respectively, with manufacturing accounting for 50.8%, 56.9% and 46.1% of the total GRP. In the more developed regions, manufacturing accounts for a relatively small proportion of GRP and pollutant emissions are relatively low. However, in less developed areas, manufacturing enterprises are still welcomed because they are needed to stimulate the local economy and create more jobs [18,19,20]. Manufacturing industries would exert more significant impacts on air pollution than other industries due to relatively intensive energy use in the production process and high pollutant emissions. Thus, the regional economic gap and difference in industrial structures will inevitably lead to varying air pollution across regions. Second, the intensities of China’s environmental regulations vary from region to region. For instance, the pollutant discharge fee rate of air pollutants in Beijing was about US$ 1.73 per unit of pollution; however, the pollutant discharge fee in the western provinces, such as Shaanxi, Ningxia, Henan, was only about US$ 0.17. Strict environmental regulations could force the emission-intensive industries to transfer from the eastern regions to the central or western regions of China with relatively less stringent environmental regulations which are regarded as “pollution paradise” [21,22,23]. Therefore, regional economic development levels and environmental regulations vary from region to region, resulting in a gap between the pollution levels. Regarding the relationship between economic growth and environmental pollution, national-level analysis provides only a general understanding of how variables are broadly related and thus provide little guidance for policymaking [24]. Understanding different patterns of economic growth and environmental pollution at the regional level and determinant factors of local pollution will help to make decisions on regional pollution control policies based on regional realities.

Different from previous studies, this paper is conducted with a primary focus on the following questions. How does the relationship between air pollution and economic growth differ by region with different stages of development? In different countries, whether there are diverse regional patterns of the relationship between air pollution and economic growth? Will the environmental quality and economic performance affect each other? The purpose of this paper is to analyze the interaction between regional economic growth and air pollution in China and South Korea (hereafter abbreviated as simply Korea). Both countries face serious air pollution problems, and the air quality of the two countries is closely related to each other. The linkages between economic growth and sulfur dioxide $\;\left({\mathrm{SO}}_{2}\right)$ emissions are estimated at the city and county level using simultaneous equation models covering 286 cities in China and 228 cities and counties in Korea during the period 2006–2016. The focus is on ${\mathrm{SO}}_{2}$ because ${\mathrm{SO}}_{2}$ emissions are recognized as a typical transboundary air pollution problem which has already induced various countries to cooperate through supranational institutions [25]. Particularly, ${\mathrm{SO}}_{2}$ is one major pollutant that poses significant risks in many developing countries undergoing a process of industrialization. ${\mathrm{SO}}_{2}$ pollution causes severe respiratory problems and significant ecosystem degradation due to acid rain formation [26,27]. Because this paper aims to examine the regional differences rather than the overall characteristics at the national level, the division of the region is a key point of this paper. To explore whether there are diverse regional patterns in different countries with respect to the relationship between air pollution and economic growth, this paper introduces two models of each country: metropolitan areas and non-metropolitan areas in China and Korea. Because of both geographic and regional endowment differences, economic development is quite uneven among different regions in China. Therefore, this paper divides China into five economic regions: eastern China, central China, northwest China, southwest China, and northeast China. The rest of this paper is organized as follows: Section 2 is a review of the literature review. Section 3 introduces the method and data source. Section 4 describes estimation results. Section 5 discusses the policy implications. The last section summarizes the conclusions.

## 2. Background

The most widely used method for analyzing the relationship between economic growth and environmental pollution is the EKC hypothesis. After the first empirical EKC study of Grossman and Krueger [1], Panayotou [2] called the inverted U-shaped pattern an Environmental Kuznets Curve after the original Kuznets curve, which describes an inverted U-shaped relationship between income and income equality Kuznets [28]. The EKC argues that in the early stage of economic development, the quality of the environment tends to decline until the average income reaches a certain level in the development stage and then improves. The validity of the environmental Kuznets curve hypothesis has been widely examined by different studies over the years. The conclusion is various because of different choices of types of data, pollutant, country and approach (as shown in Table 1). There were a few criticisms on the EKC hypothesis. First, the evidence in favor of an inverted U-shaped relation is not robust and the locations of the turning points are sensitive to both slight variations in the data and to reasonable permutations of the econometric specification [29]. Second, the empirical evidence supporting EKC crucially depends on the selected pollutant, the sample composition and the period considered [30].

Based on the EKC hypothesis, many other studies believed that the relationship between economic growth and environmental pollution is N-shaped [1,15,16,17]. Compared to the inverted U-shaped pattern, the N-shaped pattern implies that in the early stages of economic development, environmental pressure tends to rise with economic growth and then decline but rises again after reaching a critical level of economic development. Leib [15] pointed out that an N-shaped pattern exists due to the external shocks, internalization of the pollution externality, the exhaustion of abatement opportunities and decreasing returns to scale of abatement technology. With continuous economic growth, the environmental carrying capacity reaches its optimal level, and technological progress also reaches the saturation point and thus further increase in production cannot mitigate the adverse impact on the environment.

One of the different views on the EKC hypothesis is that there is a monotonically increasing curve that exists between pollution and growth [5,6,7,8,9,10]. Holtz-Eakin and Selden [5] examined the relationship between economic development and carbon dioxide emissions using the global panel data of 130 counties. They suggest that global carbon dioxide emissions growth will continue to grow at an annual rate of 1.8% in the foreseeable future and continued increase in emissions is due to the fastest growth in output and population in low-income nations. Ang [7] provided evidence for a robust long-run relationship between pollutant emissions and output in France from 1960 to 2000. The results showed that in the long run, output growth can promote both ${\mathrm{CO}}_{2}$ emissions and energy consumption. Using the time-series data from 1960 to 2005, Halicioglu [8] concluded that income was the most significant variable to explain the ${\mathrm{CO}}_{2}$ emissions in Turkey, followed by energy consumption and foreign trade. Chandran and Tang [9] found that the inverted U-shape EKC hypothesis is not applicable to Indonesia, Malaysia, and Thailand. The relationship between ${\mathrm{CO}}_{2}$ emissions and income tends to be linear in Indonesia and a normal U-shaped curve in Malaysia and Thailand. Al-Mulali et al. [10] revealed that the EKC hypothesis does not exist because the relationship between GRP and pollution is a monotonically increasing curve in both the short and long run in Vietnam during the period 1981–2011.

Another point of view contrary to the EKC hypothesis provides evidence in support of a U-shaped relationship between pollution and economic growth, indicating that pollution emission decreases with economic growth initially and then increase. Moomaw and Unruh [11] concluded that there is a U-shaped and N-shaped relationship between ${\mathrm{CO}}_{2}$ emissions and income that exist in 16 OECD (The Organisation for Economic Co-operation and Development) countries in the period from 1950 to 1992. They indicated that it is misleading to interpret EKC results as a process of income growth that all countries must pass through. Kaufmann et al. [12] indicated a U-shaped relation between the GRP per capita and the atmospheric concentration of sulfur dioxide for 23 counties between 1974 and 1989. The concentration of sulfur dioxide tended to decrease as per capita GRP rises from $ 3000 to $ 12,500 but after that the concentration of ${\mathrm{SO}}_{2}$ increased. Dinda et al. [4] found a U-shaped shift between per capita income and the annual mean concentration of suspended particulate matter and ${\mathrm{SO}}_{2}$ in 49 cities of 33 countries from 1979 to 1990. They pointed out that without environmental degradation, further increases in income cannot be because of the technical limits of industrial pollution control. In addition, Ozcan [13], Chandran and Tang [9] and Wang et al. [14] also found more similar cases of U-shaped relationship between ${\mathrm{CO}}_{2}$ emissions and economic growth in the Middle East and Asian countries. Table 1 shows the empirical research results published between 1995 and 2017. The EKC hypothesis was tested using different pollutants by variable economic indicators, such as energy consumption, gross domestic product, trade openness, industrial output, urbanization, population density, and foreign direct investment.

Although many researchers have carried out key studies on the relationship between economic growth and air pollution, most have focused on the overall characteristics of air pollution at the national level, using cross-country data or provincial level data of an individual country. In fact, due to regional heterogeneity, different countries have diverse patterns of economic growth and air pollution at the regional level. Using a panel data set of Malaysian states, Vincent [31] found that the EKC hypothesis between the emission of six pollutants and income was not fulfilled. The pollution-income relationships showed different trends of different pollutants because of different characteristics of the processes generating pollution, natural resource endowments, shifting patterns of population, and the impacts of environmental policies. List and Gallet [32] and Milimet et al. [33] used the same data set state-level panel data from the United States from 1929 to 1994 to examine the relationship between air pollutants ${\mathrm{SO}}_{2}$ and ${\mathrm{NO}}_{2}$ and per capita income. The results provided evidence that an inverted U-shape characterizes the relationship between per capita emissions and per capita incomes at the state level, but differences in the level of the turning points across states. Park and Lee [34] explored the relationship between economic growth and air pollution by using the annual panel data of 16 metropolitan areas in Korea from 1990 to 2005. The results showed that GRP is negatively related to air pollution caused by ${\mathrm{SO}}_{2}$, failing to verify an inverted U-shaped nor N-shaped curves. In the case of China, Jiang et al. [35] found an inverted U-shaped relationship between per capita income and per capita emissions for waste gas emissions from fuel burning and wastewater, while a U-shaped relationship for waste gas emissions from production using provincial panel data from 1985 to 2005. They concluded that the less developed central and western regions appear to have turning points occurring at lower income levels than the developed coastal region. Song et al. [36] examined the relationship between waste gas, wastewater, and solid waste pollution and economic growth from 1985 to 2005 using provincial data in China and found that all three pollutants showed an inverted U-shaped pattern. They also found that only a few high-income regions have reached the stage of environmental improvement, while most provinces have more severe environmental degradation. Wu et al. [37] analyzed the impacts of various carbon emission factors in the four classified regions, namely, a high economy and high carbon intensity region, a high economy and low carbon intensity region, a low economy and low carbon intensity region, and a low economy and high carbon intensity region. They concluded that capita carbon emission increases monotonically with per capita GRP in all regions, and the most significant factor of emission in four regions is the industrial structure, energy intensity, population size and per capita GRP, respectively.

These regional analyses show that there are multiple patterns of pollution-growth relationships across regions, and it is valuable to compare regions with different economic levels. This paper differs from previous studies in two ways. First, a cross-country comparative perspective is adopted to examine the relationship between air pollution and economic growth at the regional level. Compared with single-country research, this paper applies the multinational analysis of two Asian countries facing severe air pollution problems, namely China and Korea, to explore whether exists a different regional pattern among counties. Second, unlike most provincial-level studies, this paper uses city-level data and concern on the relationship between air pollution and economic growth not only among regions at different stages of development but also among cities at different scales. Previous regional studies in China generally consider the differences between eastern, central, and western China. Based on existing regional research, this paper adopts a more detailed regional division method. In addition, this paper also focuses on the regional difference between metropolitan and non-metropolitan areas.

## 3. Methods

### 3.1. Model Specification

**Figure 1:**
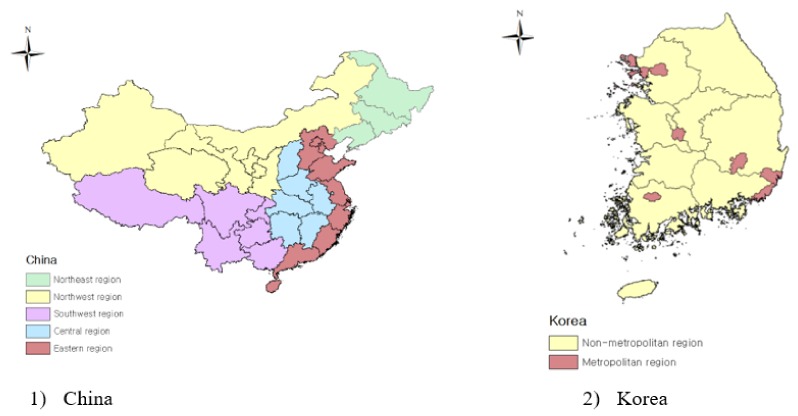
Regional Division of Two Counties.

This paper uses the simultaneous equation model (SEM) methodology to analyze the relationship between regional economic growth and air pollution covering 286 cities in China and 228 cities and counties in Korea during the period of 2006–2016 (Figure 1). An important criticism of some existing empirical EKC studies is the failure to consider the feedback effect of pollution on economic growth. Perrings [38] proposed that the economy and its environment are jointly determined. It is inappropriate to estimate a single equation model assuming unidirectional causality from economy to environment. Barbier [39] found clear evidence that in the early stages of development in many developing countries, when the environment deteriorated, rapid growth attempts could be counterproductive and unsustainable. Pollutant emissions may limit the supply of environmental inputs through environmental degradation, or they may reduce workday losses due to health problems caused by pollution and reduce production. Therefore, it is more appropriate to analyze the relationship between environmental pollution and economic growth by SEM which simultaneously estimates the parameters of the whole system.

To handle the potential endogeneity caused by the bilateral causality between economic growth and air pollution, this paper applies the SEM to test the relationship between per capita GRP and the annual emission of ${\mathrm{SO}}_{2}$ using the Three-Stage Least Squares (3SLS) method (the Two-Stage Least Squares (2SLS) method developed by Theil [40] is the basic approach for the SEM. 3SLS estimation introduced by Zellner and Theil [41] is more advanced than 2SLS because 3SLS takes into consideration the contemporaneous correlation of disturbances across the equations in the SEM. In addition, as Kennedy [42] stressed, 3SLS is generally believed to be more consistent and asymptotically more efficient; therefore, it is preferred to 2SLS if the disturbances of the separate equations are correlated). In this paper, the choice of control variables is based on evidence from the existing literature. Extensive empirical studies have attempted to analyze the causal relationships between pollutant emissions, energy consumption and economic growth [7,43,44]. Kraft and Kraft [45] proposed that the imbalance of industrial structure had a significant impact on the relationship between power consumption and economic growth. Many studies have confirmed that the impact of industrial structure is significant [43,46,47,48]. Therefore, energy consumption and industrial structure are also tested as the impact factors of the relationship between growth and pollution. Before estimating the basic model, this paper tests the cross-sectional dependence, followed by panel unit root and cointegration tests for these variables in all models. Relying on the assumptions of cross-sectional independence may lead to inefficient and inaccurate estimation results if the panel data are cross-sectionally dependent. Therefore, this paper uses the CD (Cross-sectional Dependence) test developed by Pesaran [49] to the analyzed variables to investigate whether each panel data is cross-sectionally independent. The CD test strongly rejects the null hypothesis of no cross-sectional dependence (Table A1). To cover this issue, this paper applies the UO (Ucar and Omay ) nonlinear unit root test [50], which allows for cross-sections dependence and nonlinearity. Ucar and Omay [50] propose a nonlinear panel unit root test by combining the nonlinear framework in Kapetanios et al. [51] with the panel unit root testing procedure of Im et al. [52]. The test investigates the unit root null against the alternative hypothesis that at least one individual series follow a nonlinear stationary process. The nonlinear cointegration tests could be fulfilled through investigating the unit root for the residuals based on the UO method.

The results of the panel unit root tests (Table A2) show that the original series are non-stationary sequences, and all the variables are first-order difference stationary. The UO nonlinear test refuses the null of no cointegration at the 1% significance level indicating that the null hypothesis of no cointegration can be rejected and providing support for the long-term relationships among study variables (Table A3):

(1) $$\ln\;GRP_{i,t}=\beta_{0}+\beta_{1}\times \ln EMP_{i,t}+\beta_{2}\times \ln EC_{i,t}+\beta_{3}\ln IS_{i,t}+\beta_{4}\times \ln Emission_{i,t}+\varepsilon_{1,i,t}$$

(2) $$\ln\;Emission_{i,t}=\lambda_{0}+\lambda_{1}\times lnGRP_{i,t}+\lambda_{2}\times lnGRP_{i,t}{}^{2}+\lambda_{3}\times \ln P_{i,t}+\lambda_{4}\times \ln IS_{i,t}+\lambda_{5}\times \ln EC_{i,t}+\varepsilon_{2,i,t}$$

$GRP_{i,t}\;$: Real GRP per capita of city *i* in period *t*

$Emission_{i,t}$: Per capita ${\mathrm{SO}}_{2}$ emissions of city *i* in period *t*

$IS_{i,t}$: proportion of output value of the manufacturing industry in GRP of city *i* in period *t*

$EC_{i,t}$: Electricity consumption of city *i* in period *t*

$P_{i,t}$: Population density of city *i* in period *t*

$EMP_{i,t}$: The number of employed people of city *i* in period *t*

$\varepsilon_{i,t}$: Error term

### 3.2. Data Description

In the case of China, the empirical examination of the relationship between regional economic growth and regional characteristics of air pollution is conducted at two geographic scales. This paper divides China into five regions: eastern China, central China, northwest China, southwest China, and northeast China (as shown in Figure 1) based on the classification by the National Bureau of Statistics of the Republic of China [53] (the regional division method is based on the National Bureau of Statistics of the Republic of China [53]. The 10 provinces (cities) in the east include Beijing, Tianjin, Hebei, Shanghai, Jiangsu, Zhejiang, Fujian, Shandong, Guangdong, and Hainan; the six central provinces include Shanxi, Anhui, Jiangxi, Henan, Hubei, and Hunan; and the 12 western provinces (districts and cities) including Inner Mongolia, Guangxi, Chongqing, Sichuan, Guizhou, Yunnan, Tibet, Shaanxi, Gansu, Qinghai, Ningxia and Xinjiang; the three northeastern provinces include Liaoning, Jilin and Heilongjiang. This paper divides the western region into two regions: southwest and northwest). Next, in order to test whether there are different regional patterns of pollution-economic growth relationship across countries, this paper introduces two models of each country: metropolitan areas and non-metropolitan areas in China and Korea. In China, the metropolitan areas consist of 19 first-tier cities and 30 second-tier cities, and non-metropolitan areas are defined as the rest 237 third-fourth-fifth-tier cities. Following Wang et al. [54] and Yang and Dunford [55], this paper uses the city-tier classification method which is most commonly used for city ranking in China in recent years (This method was originally published by Yicai Global and the National Bureau of Statistics of the Republic of China [56] also uses the same city classifications to conduct National Residential Sales Price Surveys. First-tier cities cover 19 cities, including 4 municipalities (Beijing, Shanghai, Tianjin and Chongqing) and 15 provincial capital cities (New first-tier cities). These cities have a strong economic base, a population of more than 6 million and abundant educational resources, advanced technology, and convenient transportation. Second-tier cities cover 30 medium-sized prefecture cities equip with a good economic base and a population of 4–7 million, including the most eastern coastal open cities and few capital cities in the central and western China. Third-fourth and fifth-tier cities cover the rest 237 cities, mainly composed of small-sized cities located in central and western China equip with the relatively underdeveloped economic condition and a population below 5 million.

In Korea, metropolitan areas cover 74 counties and cities in seven major cities, including Seoul, Busan, Daegu, Incheon, Gwangju, Daejeon, and Ulsan. Non-metropolitan areas are composed of the remaining 154 cities and counties (Figure 1). The Chinese data comes mainly from the City Statistical Yearbook of China from 2006 to 2016, published by the National Bureau of Statistics of China. The Korean data is collected from the Korean Statistical Information Service operated by Statistics Korea and the National Institute of Environmental Research. Because the absence of per capita GRP data at the county level for Seoul Special City, this paper calculated the GRP in every district of Seoul by multiplying the proportion of employment in each sector by the total added value. Table 2 reports summary statistics of the variables that are used in the estimation of region-specific models.

### 3.3. Robustness Tests

It is necessary to check how robust the analysis is because the EKC hypothesis is assessed and tested in various directions, including alternative functional forms, different econometric methods, the inclusion of additional explanatory variables [57,58,59]. First, this paper tested the relationship between per capita GRP and three emission indices, including per capita ${\mathrm{SO}}_{2}$ emission (Total ${\mathrm{SO}}_{2}$ emissions emissions/population), ${\mathrm{SO}}_{2}$ emission intensity (Total ${\mathrm{SO}}_{2}$ emissions/industrial output value) and ${\mathrm{SO}}_{2}$ emissions density (Total ${\mathrm{SO}}_{2}$ emissions/land area). The results show that the relationship between the three indexes and per capita GRP shows the same patterns by region, although the pollution index is changed, the parameter symbols of ln(GRP) and its quadratic term are consistent in all panel models. Because only the per capita ${\mathrm{SO}}_{2}$ emission index is significantly correlated with the per capita GRP variable and its quadratic term under the significant level of 10% in all models, we only reported the results of the models using the index of per capita emission. Second, for choosing the functional form, this paper uses T-test to check the statistical significance quadratic term and cubic terms of per capita GRP and omit the cubic terms. Because all coefficients are insignificant in the cubic estimation, but coefficients in the quadratic estimation are highly significant, we only consider the quadratic terms in our model. The third step is to choose the estimation method. After examining the endogeneity of the explanatory variables by Hausman’s test, the per capita GRP variables and its quadratic terms were rejected as exogenous in the simultaneous equation model. Therefore, this paper uses the Three-stage least squares method (3SLS) and the interaction terms of exogenous variables and their quadratic terms were chosen as the instrumental variable for the per capita GRP and its quadratic term. Besides, this paper applies the over-identification test and weak instrument test to make sure all the selected instruments are valid. Through the three dimensions of robustness tests above, this paper confirms the final model which tests the relationship between the per capita ${\mathrm{SO}}_{2}$ emission index and per capita GRP and its quadratic term. Moreover, the Mann-Whitney U test was used to compare the differences in the levels of GRP per capita and pollution emission levels across metropolitan and non-metropolitan areas in both Chinese case and Korean case. In both counties, the results of the Mann-Whitney U test (as shown in the Table A4) show that the null hypothesis of no statistical differences is rejected with the value of significance (0.001) is much smaller than α (0.05). It can be concluded that there are significant differences in the levels of economic growth and pollution emission between metropolitan areas and non-metropolitan areas in China and Korea.

## 4. Results

**Figure A1:**
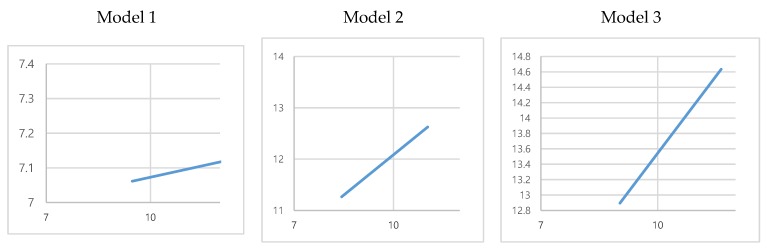

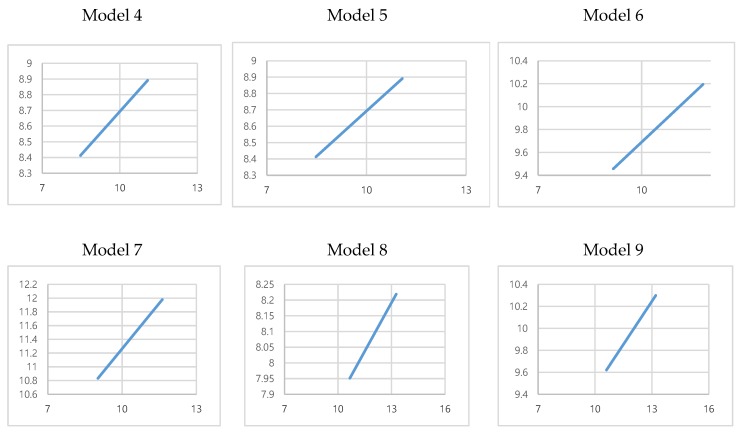
Growth-Pollution Patterns. Notes: The X-axis is per capita SO_2_ emission (ton), Y-axis is GDP per capita ($).

**Figure A2:**
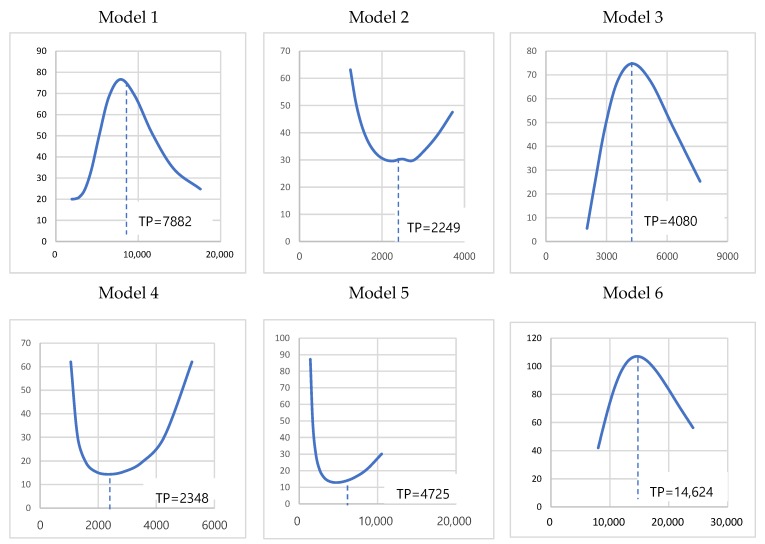

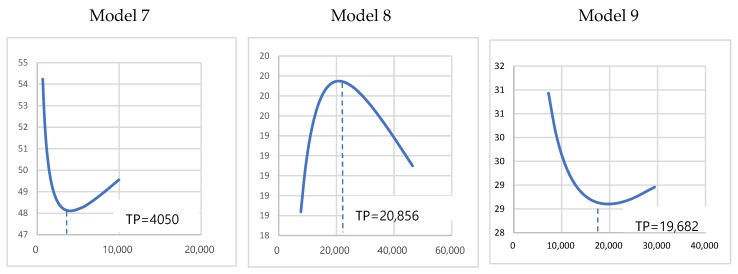
Pollution-Growth Patterns. Notes: The X-axis is GRP per capita ($), Y-axis is per capita SO_2_ emission (ton).TP means the level of GRP per capita at the turning points of each region.

Table 3 presents the empirical results of the SEM. The results show that not only does regional economic growth have a significant impact on air pollution, but also the impact of air pollution on regional economic growth is significant (Figure A1 and Figure A2). More importantly, the relationship between air pollution and economic growth varies in different regions. From the respective of equations of GRP per capita, the growth of GRP per capita in all models is positively correlated with per capita emission. The economic growth was much more associated with the increase in pollutant emission of less developed regions than developed regions. For instance, over the last decade, a 1% increase of per capita emission could result in a 0.02% increase in the GRP per capita on average per year in east China, while 0.52%, 0.67% and 0.28% for central, northwest and northeast China. At the same time, a 1% increase in per capita emission of non-metropolitan areas resulted in 0.44% and 0.26% of growth in per capita GRP in China and Korea, respectively, which is 0.36% and 0.16% larger than that in the metropolitan region. It indicates that in both countries, per capita emission has a stronger effect on economic growth in non-metropolitan areas than metropolitan areas. This result implies that the development of manufacturing industries, in particular, pollution-intensive industries in non-metropolitan areas has contributed more to economic growth than metropolitan areas.

**Figure 2:**
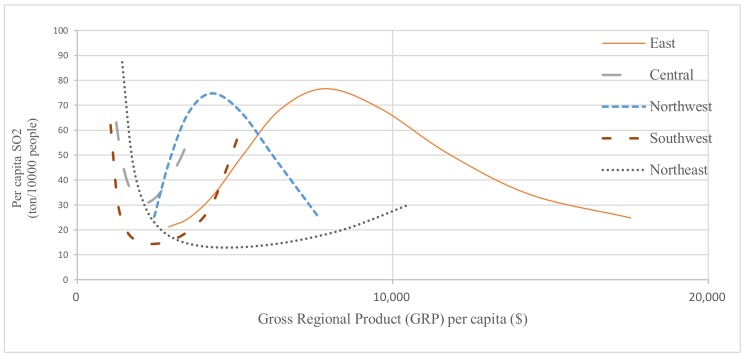
Pollution-Growth Patterns at Five Regions in China.

From the respective of equations of per capita emission, the results illustrated with the Table 3 models (1)–(5) show that different patterns were found in China at the regional level. Only in the east and northwest region, the sighs of coefficients of the GRP per capita and its square terms are negative and positive, respectively, which suggests that the regional pollution level tended to follow an inverted U-shape pattern in the period 2006–2016. Such a pattern implies that pollutant emissions decrease with economic growth after experienced a stage of environmental degradation in the two regions (Figure 2). According to the China Statistical Yearbook, the proportion of output values of manufacturing to total GRP from 2006 to 2016 decreased by 19.77% in the eastern region and 17.04% in the northwest respectively. However, the decline in the proportion of manufacturing in total GRP only realized 8.20% in the southwest and 7.76% in the central region. According to the National Bureau of Statistics of the Republic of China, from 2006 to 2016, the proportion of the total output of manufacturing to total GRP dropped from 46.58% to 36.62% in the eastern region and decreased from 39.47% to 28.94% in the northwest region. In the southwest, the proportion decreased from 36.10% to 30.84%; the central region decreased from 42.08% to 38.46%, and in the northeast region decreased from 44.09% to 30.77%. In the east region and northwest region, energy-intensive industries gradually concentrated in the sectors with lower energy intensity. Another reason for the declining trend in the developed eastern regions is strict environmental regulations for polluting enterprises. As mentioned before, the pollutant discharge fee rate of air pollutants in the eastern region, such as Beijing, was about ten times higher than that in the western provinces. Stricter environmental regulations in the eastern region have led to the booming of environmentally friendly enterprises and innovations in industries.

However, the relationship between air pollution and economic growth in the rest of China was different. The results of models (2), (4) and (5) demonstrate a U-shaped curve relationship in southwest, central and northeast region indicating that the pollutant emissions increase with economic growth after the per capita GRP level reaches a certain level. To eliminate the economic gap in China, the central government implemented the Western Development Strategy and Rise of Central China Plan from 2000 to 2004, and a large amount of investment promoted the regional output, effectively increasing the GDP of the western and central region.

Moreover, with the adjustment of China’s economic development strategy in recent years, the western region has received less attention than before, and environmental problems have become more and more serious [60]. In order to pursue economic development goals and attract investment, local governments have reduced environmental protection investment and weakened environmental regulations. As a result, the problems of environmental pollution are increasingly severe with economic growth. In particular, the proportion of output values of manufacturing to total GRP declined by 28.44% in the northeast from 2006 to 2016. Even though there is a U-shaped curve, the average GRP per capita level was still at the declining stage of the U curve indicating that the pollution emission level had been decreased with economic growth in the period of 2006-2016 in the northeast. Northeast is China’s largest old industrial base.

Since the implementation of the revitalization policy of the old industrial bases in the northeast in 2003, the proportion of ${\mathrm{SO}}_{2}$ high-load sectors in total industrial output declined from 23.87% in 2005 to 20.36% in 2013 due to the transformation of the regional industrial structure and the technological progress [61]. Therefore, adjustment and optimization of the industrial structure were found to be an effective mean to reduce the air pollution.

The results of models (6)–(9) show that the pattern of economic growth and pollutant emissions among metropolitan and non-metropolitan areas is reversed in both countries. From 2006 to 2016, there was a common inverted U-shaped pattern is found in metropolitan areas of China and Korea. Overall, it indicates that in metropolitan areas of both countries, the pollutant emission decreased with economic growth. Conversely, in non-metropolitan areas, the U-shaped pattern implied that the pollutant emission overall increased with economic growth. Such a different result confirms that the impacts of economic growth on pollutant emission tend to be spatially heterogeneous among cities with different scales in China and Korea.

The different patterns between metropolitan and non-metropolitan areas in both two countries could be explained by the more stringent pollution regulation in metropolitan areas. With the rapid urbanization, air pollution problems have been increasing concern in China. In order to better improve the regional air quality throughout metropolitan areas, the Chinese government has implemented a series of national control policies to reduce the emissions of air pollutants since 2005. For instance, the Ministry of Environmental Protection has issued the action of “Joint Prevention and Control of Air Pollution” in 2011, which aims to establish a joint prevention and control system and effectively improve the regional air quality. It was firstly implemented in three key regions which cover the major metropolitan areas in China, including the Beijing-Tianjin-Hebei Region, the Yangtze River Delta and the Pearl River Delta during the period of the 12th Five-Year Plan (2011–2015). The decrease in ${\mathrm{SO}}_{2}$ from 2006 to 2016 in the metropolitan areas reflects the success of China’s air pollution control program.

In the case of Korea, the government legislated a special act named “Improvement of Air Quality in Seoul Metropolitan Areas (SMA, areas including Seoul and Incheon metropolitan cities, Gyeonggi province)” in December 2003 to improve the air quality in SMA. The main focus of the air quality improvement plan for SMA (2005–2014) was to regulate the total amount of emissions in the workplace, to supply low-emission vehicles, and to strengthen gas emission management regulations [62]. After the implementation of the first phase of an air quality improvement plan, the annual concentration of main pollutants has significantly decreased in Seoul and Incheon until 2013 [63]. Again, the government adopted the second phase of the air quality improvement plan from 2015 to 2024 for SMA [64].

**Figure 3:**
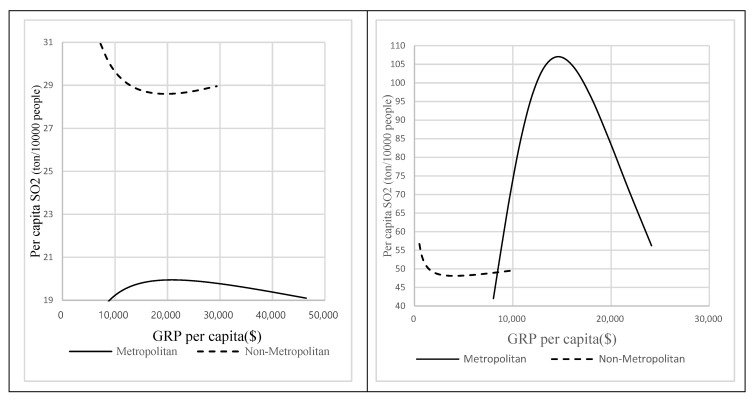
Pollution-Growth Patterns at Different City Scales in Two Countries.

There is still a large gap between the overall emission levels of China and South Korea, although pollutant emissions in both metropolitan areas show a downward trend. Figure 3 shows that China not only has a higher overall level of pollutant emissions than Korea, but also a larger disparity in regional economic levels. For instance, the average GRP per capita level was found to be US $10,356 and US $3851 for metropolitan and non-metropolitan areas in China, whereas US $19,020 and US $17,779, respectively, in Korea (Table 2). Another significant difference is that the level of per capita pollutant emission in China’s metropolitan areas was still much higher than in non-metropolitan areas, while the opposite result has occurred in Korea. The overall level of pollutant emission in Korea’s metropolitan areas was obviously lower than in non-metropolitan areas. This can be interpreted as that higher pollutant emission levels in non-metropolitan areas were associated with a relatively higher proportion of manufacturing industry in GRP. The average proportion of the manufacturing industries in non-metropolitan areas was 13% from 2006 to 2016, which is 5% higher than that in metropolitan areas (Table 2), indicating that more manufacturing enterprises in Korea prefer to locate in non-metropolitan areas. In China, manufacturing still occupied an important position in metropolitan areas (48%) in the last decade. In other words, although pollution levels in metropolitan areas of the two countries have shown a downward trend in the past ten years, there is still a large gap between China and Korea in terms of the overall level of pollutant emissions, especially the level of pollutant emissions of metropolitan areas.

From the respective of determinants of pollutant emission, this paper compares the elasticities of pollutant emission with respect to energy consumption, industrial structure and population density based on the reduced form of simultaneous equation models. After controlling the effect of economic growth, the main determinants of air pollution are energy consumption and industrial structure in metropolitan and non-metropolitan areas, respectively. For instance, with a 1% increase in the proportion of manufacturing industry output in GRP, industrial ${\mathrm{SO}}_{2}$ emission in non-metropolitan areas increased by 0.04% and 0.30% in China and Korea, larger than that in metropolitan areas (0.01% and 0.15%). It suggests that industrial structure has a relative stronger effect on pollutant emission in non-metropolitan areas than metropolitan areas. On the other hand, in the case of Korea, with a 1% increase in energy consumption, industrial ${\mathrm{SO}}_{2}$ emission in non-metropolitan areas increased by 0.38% and 0.17% in metropolitan areas and non-metropolitan areas, respectively. In the case of China, a 1% increase in energy consumption was associated with a 0.48% increase in pollutant emission of metropolitan areas and a 0.03% increase in non-metropolitan areas. The results find that impacts of energy consumption on pollutant emission were larger in metropolitan areas than non-metropolitan areas in both China and Korea. In addition, the results found that there was a negative effect of population density of the pollutant emission of most models; however, the relationship between air pollution and population density was uncertain for the northwest region in China and non-metropolitan areas in Korea.

## 5. Policy Implications

According to the analysis results of this paper, the policy implications could be summarized from the following three respects: the national level, the regional level, and the city level. The inverted U-shape patterns of the two countries suggest that pollution levels in metropolitan areas have shown a downward trend in recent years, whereas there is still a large gap between the overall level of pollutant emissions of China and Korea. This gap could be attributed to the difference in economic development stages and the implementation of environmental policies of the two counties. Si et al. [65] proposed that many environmental policies in China are communicated in the form of government documents lacking clear legal provisions and regulations. The update of existing environmental laws and regulations is also relatively slow, and environmental standards cannot adapt to changing environmental issues. In Korea, environmental policy tools are set up in a way that not only controls the whole process, also controls before and after the event; besides, reasonable environmental laws and regulations, and timely updating mechanism provide a guarantee for the effective implementation of environmental policy tools. Drawing on the experience of South Korea, if the Chinese government pays more attention to improving the environmental legal system, it will have an effective impact on improving air pollution, including formulating various market-oriented policy tools, such as environmental taxes and fees, and a more detailed emissions trading market, and encourage enterprises to establish their own pollution monitoring systems.

Different from the previous regional research suggesting that there was a similar pattern in the relationship between air pollution and economic growth across regions in China [35,36,37]. This paper uses a more detail regional division method and finds that diverse patterns exist in five regions of China at different stages of development from 2006 to 2016. The experience of the development model of the eastern region can provide inspiration for the balanced growth and pollution of the southwest and central regions from the following three aspects. First, as air pollution levels in the central and southwest regions deteriorate with economic growth, the government needs to pay more attention to change traditional high energy-consuming and high-polluting production methods of the local energy-intensive industries to reduce pollutant emissions. At the same time, it is necessary to strengthen the improvement of production technology, promote the innovation and promotion of clean production technology. Second, differentiated regional environmental policies should be designed for different stages of economic development. In the central and southwestern regions, the main sources of air pollution are the emissions of industrial pollutants. The environmental regulations for polluted enterprises could be further strengthened in areas where the heavy industries and highly polluting industries are densely located. The appropriate environmental regulations could help to accelerate industrial upgrading and economic transition to a more sustainable style. Besides, the strong pollution control policy is still needed even there is a clear downward trend of pollutant emission in the eastern, northeastern and northwest region. Third, increase investment in pollution control for the southwestern and central region. The U shape pattern of these two regions means that a large amount of environmental pollution generated in the process of pursuing rapid economic growth. Even though the local government has started to increase investment in pollution control, but it has not played a substantial role in recent years [60]. In the western and central region, it is urgent to increase financial support and arrange for specific financial resources to invest in environmental protection, especially the improvement of air quality, and strengthen supervision and management of investment in environmental protection.

The gaps between China’s metropolitan areas and non-metropolitan areas in the terms of both economic development level and pollutant emission level are much larger than those of Korea. To improve regional air quality, the actual situation and major determinants of air pollution in both urban and non-urban areas in China need to be considered. Since the major determinant of air pollutant emissions in metropolitan areas is the energy consumption, it is crucial to develop new energy industries to reduce pollutant emissions. It not only relies on the government’s financial subsidies, tax incentives and other incentive policies, but also promoting the technological progress and industrial upgrading of renewable energy through the market mechanism. In the relatively backward non-metropolitan areas, it is important to continuously adjust the industrial structure by encouraging the development of the service industry, as well as promote the optimization of the internal structure of the secondary industry and encourage the development of low-pollution and low-energy-consuming industries.

## 6. Discussion and Conclusions

The purpose of this paper is to analyze the relationship between regional economic growth and air pollution in China and Korea to explore whether there are diverse regional patterns of different countries. The major findings of this paper are that regional differences existed in the relationship between air pollution and economic growth in China and Korea. In both countries, an inverted U-shaped pattern was found in metropolitan areas while a U-shaped pattern exists in non-metropolitan areas. Moreover, there exist different patterns in five regions of China, which are influenced by different levels of economic development and different regional environmental policies. There is still a large gap between the overall emission levels of China and Korea although the emissions of pollutants in metropolitan areas of both countries have shown a downward trend in recent years. The level of pollutant emissions of China’s metropolitan areas is much higher than in non-metropolitan areas, while the opposite result has occurred in Korea.

This paper contributes to the literature from three respects. First, in contrast to the extensive literature on the relationship between air pollution and economic growth, this study reveals the importance of regional-level research. Based on the existing regional studies, this paper attempts to adopt a more detailed regional division method and points out that the patterns of air pollution vary significantly between regions, filling a gap in relevant research fields at the regional level. Second, this paper focuses on the differences between cities of different sizes. Different patterns of pollution development in metropolitan and non-metropolitan areas are compared. In addition, this paper makes a comparison between two Asian countries, China and South Korea, which are facing serious air pollution problems as well as large regional differences in economic development levels. One of the limitations of this paper is that it only focuses on ${\mathrm{SO}}_{2}$, leaving room for further study of other pollutants in the future. Industrial development in different regions is closely related to local natural resources, and our analysis may ignore the impact of production activities in some pollution-intensive industries with other pollutants as the main pollutants on air quality. For further research, more diverse pollution phenomena need to be paid attention to, including other air pollutants, water pollution, and solid pollution. This paper mainly analyzes the impact of economic growth, industrial structure, energy consumption and population factors on air pollution in the two countries. However, the environmental policies of the two countries also play a very important role in regional air pollution. Since it is difficult to develop a common policy variable available for both countries, more policy factors have not been taken into account. In future research, it is necessary to continue to pay attention to this direction of research. In addition, the limitation of the methodology in this paper is that, due to the limitation of data, the model only considers short-term analysis and cannot establish the long-term dynamic analysis model.

## Figures and Tables

**Table 1:** Reviews on the Relationship between Economic Growth and Pollution.

Patterns	Authors	Dependent Variables	Independent Variables
Monotonic rising curve	Holtz-Eakin and Selden [5], Dasgupta et al. [6], Ang [7], Halicioglu [8], Chandran and Tang [9], Al-Mulali, et al. [10]	Annual emissions of ${\mathrm{CO}}_{2}$	Gross Regional Product (GRP) per capita and square, Energy Consumption, Output, Foreign Direct Investment (FDI), Transport energy consumption, Labor Force, Exports and Imports
Inverted U-shaped	Grossman and Krueger [1], Panayotou [2]	Annual emissions of ${\mathrm{NO}}_{2}$, ${\mathrm{SO}}_{2}$, suspended particulate matter	GRP per capita and square, Population density, Industry Shares in GRP, Trade Intensity
U-shaped	Moomaw and Unruh [11], Kaufmann et al. [12], Dinda et al. [4], Ozcan [13], Chandran and Tang [9], Wang et al. [14]	Annual emissions of ${\mathrm{CO}}_{2}$, ${\mathrm{SO}}_{2}$, suspended particulate matter	GRP per capita, Population growth, Spatial intensity of economic activity, Energy consumption, FDI, Transport energy consumption
N-shaped	Grossman and Krueger [1], Aslanidis and Xepapadeas [16], Babu and Datta [17]	Annual emissions of ${\mathrm{CO}}_{2}$, ${\mathrm{NO}}_{2}$, ${\mathrm{SO}}_{2}$, Soil quality index, Water quality index, Air quality index	GRP per capita, GRP, GRP square, GRP cubic, Temperature, Import shares, Share of the tertiary industry, Environmental degradation index and Population

**Table 2:** Data Analysis.

	China														Korea			
	East		Central		Southwest		Northwest		Northeast		Metropolitan		Non-Metro		Metropolitan		Non-Metro	
	Mean	SD	Mean	SD	Mean	SD	Mean	SD	Mean	SD	Mean	SD	Mean	SD	Mean	SD	Mean	SD
GRP	6182	0.801	3244	0.642	3944	0.951	2536	0.654	4551	0.657	10,356	0.586	3,851	0.712	19,020	0.632	17,779	1.236
EMISSION	42	1.198	37	0.858	37	1.200	30	1.186	31	0.958	70	0.408	34	0.559	9	1.653	33	2.066
EMP	54	0.855	33	0.623	19	0.741	24	0.797	30	0.619	134	0.867	31	1.459	367	0.239	314	0.563
IS	36	8.466	42	9.875	37	14.470	41	9.697	40	13.088	48	7.398	49	11.939	8	1.098	13	1.300
EC	1173	1.132	491	1.246	622	2.065	364	1.218	995	1.181	2083	0.673	592	1.028	2569	0.961	4829	1.262
POP	54	0.606	42	0.593	10	1.009	28	0.696	16	0.719	39	1.312	28	1.476	77	1.029	10	0.967

Notes: GRP represents the GRP per capita (US$); EMISSION represents per capita emission (ton/10,000people); EMP represents employment (per 1000people); IS represents the share of manufacturing industry in GRP (%); EC represents the energy consumption (kWh/person); POP represents population density (10,000 people/km^2^).SD is the standard deviation.

**Table 3:** Estimation Results of Simultaneous Equations by Three-Stages Least Squares Method.

	China							Korea	
	East Model (1)	Central Model (2)	Northwest Model (3)	Southwest Model (4)	Northeast Model (5)	Metropolitan Model (6)	Non- Metropolitan Model (7)	Metropolitan Model (8)	Non- Metropolitan Model (9)
**Equation of GRP per capita**									
	Parameter (SE)	Parameter (SE)	Parameter (SE)	Parameter (SE)	Parameter (SE)	Parameter (SE)	Parameter (SE)	Parameter (SE)	Parameter (SE)
Intercept	6.853 *** (15.28)	2.886 *** (2.63)	1.358 (0.81)	5.762 *** (3.39)	5.123 *** (5.06)	6.224 *** (0.73)	1.170 *** (0.89)	6.285 *** (28.93)	5.838 *** (16.81)
EMP	0.233 *** (7.55)	0.0552 (111)	0.062 (0.67)	0.118 *** (1.22)	0.179 *** (2.58)	0.039 *** (0.73)	0.485 *** (13.20)	0.685 *** (16.66)	0.372 *** (2.98)
EC	0.480 *** (33.19)	0.2514 *** (10.57)	0.222 *** (8.5)	0.209 *** (7.33)	0.229 *** (6.80)	0.599 *** (16.16)	0.377 *** (19.99)	0.122 *** (7.27)	0.218 *** (6.46)
IS	−0.012 *** (-3.05)	−0.0021 (−0.43)	0.005 (0.95)	0.012 ** (2.53)	0.004 (1.47)	−0.015 (-3.05)	0.003 *** (1.43)	−0.011 (−1.02)	−0.001 (−0.04)
EMISSION	0.022 *** (0.34)	0.5231 *** (4.05)	0.669 ** (3.62)	0.184 (0.93)	0.2837 ** (2.57)	0.083 *** (0.28)	0.441 *** (3.54)	0.103 *** (7.82)	0.261 *** (4.99)
**Equation of Per capita emission**									
	Parameter (SE)	Parameter (SE)	Parameter (SE)	Parameter (SE)	Parameter (SE)	Parameter (SE)	Parameter (SE)	Parameter (SE)	Parameter (SE)
Intercept	−446.000 *** (−3.79)	125.429 *** (3.02)	−203.22 ** (−2.2)	285.475 *** (2.85)	150.181 * (1.94)	10.719 *** (0.61)	15.356 *** (0.31)	25.286 *** (4.50)	24.014 *** (0.54)
GRP	84.002 *** (3.84)	−23.915 *** (−2.87)	41.967 ** (2.27)	−57.604 *** (−2.81)	−27.563 * (−1.86)	56.317 *** (0.63)	−0.941 *** (−0.11)	1.371 *** (−4.61)	−2.336 *** (−0.65)
${\;\mathrm{GRP}}^{2}$	−3.857 *** (−3.80)	1.241 *** (2.96)	−2.051 ** (−2.23)	2.976 *** (2.86)	1.328 * (1.89)	−2.447 *** (−0.63)	0.046 *** (0.14)	−0.068 *** (10.16)	0.118 *** (0.77)
EC	−0.002 (−0.01)	0.138 *** (3.46)	0.171 ** (2.2)	0.272 *** (3.35)	0.174 *** (2.88)	−0.115 *** (−0.66)	0.023 *** (0.64)	0.785 *** (6.64)	0.632 *** (12.29)
IS	−0.006 (−0.610)	0.013 *** (4.24)	0.009 (0.7)	−0.0001 (−0.01)	−0.005 (−0.85)	0.042 *** (0.81)	0.060 *** (12.46)	0.168 *** (1.96)	0.442 *** (5.56)
P	0.577 ** (1.99)	-0.510 *** (−5.99)	−0.182 (−0.9)	0.211 ** (1.80)	0.594 *** (4.96)	−0.154 *** (−0.52)	−0.102 *** (−4.30)	−0.632 *** (10.25)	−0.026 (1.68)
R-square	0.7483	0.6680	0.5703	0.5842	0.5706	0.6844	0.5564	0.5139	0.7611
Number of Cross sections	101	88	38	46	33	49	237	74	154
Time series length: 2006–2016									
Turning point ($)	7882	2249	4080	2348	4725	14624	4050	20856	19682
Pattern	Inverted U shape	U shape	Inverted U shape	U shape	U shape	Inverted U shape	U shape	Inverted U shape	U shape

Notes: The curve shapes between per capita SO2 and GDP per capita can be determined from the signs of parameters: U-shape (If $\lambda_{1}\langle 0,\;\lambda_{2}\rangle 0$), inverted U-shape (if $\lambda_{1}>0,\;\lambda_{2}<0$). All variables are in log form *** 1% significance level; ** 5% significance level; * 10% significance level. Standard errors are in brackets.

## Appendix A

**Table A1:** Results of Cross-sectional Dependence Test.

	Panel A	Panel B
Pesaran CD	39.773 ***	23.724 ***
*p*-value	0.000	0.000
Number of observations	286	228

Notes: Panel A includes 285 cities of China, 2006–2016; Panel A includes 285 cities of China, 2006–2016. *** 1% significance level.

**Table A2:** Results of Panel Unit Root Test.

	Panel A		Panel B	
	Level	First differences	Level	First differences
EMISSION	2.930	5.003 ***	1.114	4.558 ***
GRP	2.488	9.015 ***	1.649	8.570 ***
GRP2	2.090	4.002 ***	7.173	24.447 ***
EMP	1.269	1.705 ***	5.192	5.260 ***
EC	0.930	2.347 ***	1.028	2.792 ***
IS	3.924	8.580 ***	4.641	9.025 ***
POP	2.314	3.259 ***	2.737	16.704 ***

Notes: Panel A includes 286 cities of China, 2006–2016; Panel B includes 228 cities and counties of Korea, 2006–2016. *** 1% significance level.

**Table A3:** Results of Cointegration test.

	Statistic	*p*-Value
Panel A.		
Equation of GRP per capita	0.063 ***	0.000
Equation of per capita Emission	0.236 ***	0.000
Panel B.		
Equation of GRP per capita	1.115 ***	0.000
Equation of per capita Emission	0.664 ***	0.000

Notes: Panel A includes 286 cities of China, 2006–2016; Panel B includes 228 cities and counties of Korea, 2006–2016. *** 1% significance level.

**Table A4:** Results of Mann-Whitney U-test.

	U-Value	Z-Statistics	*p*-Value
Korea			
GRP	193,144	−6.4637 ***	<0.00001
Emission	175,605	−8.6696 ***	<0.00001
China			
GRP	220,681	15.0719 ***	<0.00001
Emission	972,885	16.6324 ***	<0.00001

Notes: A value of *p* < 0.05 was considered to be significant. *** 1% significance level.
